# Effectiveness and Safety of Low-Level Red Light and Short-Wavelength Light Therapy for Myopia Control and Prevention in Children: A Systematic Review

**DOI:** 10.3390/jcm15218493

**Published:** 2026-11-01

**Authors:** Saif Hassan Alrasheed, Naveen Kumar Challa

**Affiliations:** Department of Optometry, College of Applied Medical Sciences, Qassim University, Buraydah 51452, Saudi Arabia

**Keywords:** myopia control, clinical feature, light therapy, child, follow-up

## Abstract

**Background:** Myopia is a major public health challenge, particularly among children, with prevalence increasing worldwide. Emerging light-based interventions, including low-level red light (LLRL) and short-wavelength light therapy, may be effective for myopia control. This systematic review aimed to assess the effectiveness and safety of LLRL and short-wavelength light therapy for myopia control in children. **Methods:** A systematic search was performed in PubMed, Web of Science, Scopus, Google Scholar, and EBSCO in accordance with PRISMA 2020 guidelines. Studies published between 2013 and 2026 assessing the effectiveness and safety of LLRL and short-wavelength therapies for myopia control were included. The quality of the included studies was assessed using the Downs and Black checklist. Intervention outcomes included changes in axial length (AL), spherical equivalent refraction (SER), and choroidal thickness (CT). Safety outcomes comprised evaluations of both structural and functional retinal damage, along with assessment of ocular surface complications following the intervention. The review protocol was registered in PROSPERO (with registration number CRD420261289443). **Results:** Twenty-six studies reported that LLRL therapy effectively slowed myopia progression in children; however, some retinal structural and functional changes warrant further long-term safety evaluation. LLRL significantly reduced AL elongation and SER progression, increased macular CT, and delayed the onset of myopia. Some studies indicated that LLRL therapy may achieve greater efficacy in myopia control compared with 0.01% atropine. Reported adverse events include photophobia, dry eye symptoms, altered color perception, conjunctivitis, and visual discomfort. Six studies showed that short-wavelength light therapy reduced AL elongation and induced short-term choroidal changes. No serious ocular or systemic adverse events were reported with short-wavelength light therapy; however, long-term safety requires further investigation. **Conclusions:** Light-based therapies, particularly LLRL and short-wavelength light interventions, show promise as non-pharmacological approaches for myopia control; conversely, current evidence remains insufficient to establish their long-term safety. LLRL therapy has demonstrated efficacy in reducing AL, increasing CT, and delaying SER progression. However, reported retinal changes and ocular surface symptoms underscore the importance of close monitoring, standardized treatment protocols, and long-term safety evaluation in large-scale clinical trials.

## 1. Introduction

Myopia has emerged as a global public health concern, particularly among children, with its prevalence estimated at approximately 30% worldwide and projected to rise to nearly 50% by 2050 [1,2]. Myopia is recognized as the second leading cause of global blindness, currently affecting an estimated 2.6 billion people globally [2,3]. Myopia can lead to serious ocular complications, particularly in cases of high myopia, which is associated with increased risk of cataracts, glaucoma, retinal detachment, and myopic macular degeneration. These conditions may result in irreversible visual impairment. Furthermore, the risk of these complications increases significantly with increasing severity of uncontrolled myopia [4,5]. Therefore, effective strategies to prevent myopia onset and slow its progression are urgently needed.

Current myopia management strategies include atropine, orthokeratology, multifocal soft contact lenses, and myopia control spectacles [6]. Although effective, contact lens-based interventions carry a risk of microbial keratitis, while higher concentrations of atropine may cause pupillary dilation, reduced accommodation, and blurred near vision [7]. Treatment adherence and patient dropout also remains a concern. Newer spectacle designs, including defocus-incorporated multiple segments (DIMS), highly aspherical lenslet (HAL) lenses and diffusion optics technology (DOT) lenses, have demonstrated significant efficacy with favorable safety profiles. More recently, low-level red light (LLRL) therapy has emerged as a potential alternative approach to myopia control. Overall, these therapies for controlling myopia are hindered by compliance problems, side effects, and lack of data regarding long-term efficacy [6,8]. Given the limitations of current optical and pharmacological interventions, considerable attention has been directed toward novel non-pharmacological approaches including light therapy. Recently, increasing attention has been directed towards the effects of light wavelengths rather than the light intensity on the onset and progression of myopia. Light is a form of electromagnetic radiation encompassing a spectrum of wavelengths, including infrared, ultraviolet, radio waves, and visible light [9]. Among the various light-based interventions investigated for myopia control, LLRL has attracted considerable attention because of its promising clinical outcomes. LLRL may inhibit scleral remodeling by reducing the expression of type I collagen in human fibroblasts and thereby slowing the progression of myopia [10].

In recent years, studies have focused on the use of red and ultraviolet light therapy as innovative, non-pharmacological approaches for the prevention and management of childhood myopia [8]. LLRL therapy involves the delivery of a concentrated laser beam of light, typically with a wavelength of about 650 nm, to irradiate the retina in a controlled manner [11,12]. Both LLRL and short-wavelength therapies are based on the principle that adequate exposure to bright outdoor light is a strong protective factor against the development of myopia [1,2,3,4,5,6]. Recent studies suggest that LLRL therapy may be more effective than current standard treatments in inhibiting myopia progression; however, further well-designed long-term studies are needed to confirm its clinical significance [13,14].

In addition to LLRL, other wavelengths of light also have been investigated for their potential role in myopia control and prevention. In contrast to LLRL therapy, short-wavelength interventions have been less extensively studied. Previous studies [15,16] have revealed that exposure to red, green, blue, and violet wavelengths can help inhibit myopia progression, with violet light (360–400 nm) showing the strongest effect. A previous study [17] revealed that violet light (VL) inhibited axial elongation and reduced the myopic shift in an induced-myopia model in chicks. The suppressive effect of VL on myopia progression is mediated through the maintenance of choroidal thickness, a process regulated by opsin-5 (OPN5) in the retina. OPN5 belongs to the opsin family of photoreceptors and is specifically sensitive to VL, enabling it to play a key role in retinal signaling pathways that influence eye growth [18]. These findings suggest that VL may influence ocular growth through biological mechanisms distinct from those proposed for LLRL therapy. Despite growing interest in light therapies for myopia control, the available evidence remains fragmented, and the efficacy and safety of LLRL and short-wavelength interventions have not been comprehensively synthesized. Furthermore, uncertainties remain regarding its long-term effectiveness and clinical applicability. Therefore, the present systematic review aims to assess the efficacy and safety of LLRL and short-wavelength therapies for the prevention and control of myopia in children.

## 2. Methods

### 2.1. Study Design

This systematic review was conducted in accordance with the Preferred Reporting Items for Systematic Reviews (PRISMA 2020) guidelines [19]. The PRISMA 2020 checklist is provided in the Supplementary Materials (Table S1). In addition, the review protocol was registered in the International Prospective Register of Systematic Reviews (PROSPERO; registered 20 January 2026) under the number CRD420261289443, which enhances accountability and reproducibility. A comprehensive literature search was conducted using several electronic databases to ensure the inclusion of all related studies. The databases searched were Web of Science, Scopus, PubMed, Google Scholar and EBSCO. This multi-database approach was designed to maximize retrieval of relevant studies and reduce the risk of missing eligible evidence. A literature search was conducted to include a broad spectrum of relevant evidence using original studies published between 2013 and 2026. The search period (2013–2026) was selected to focus on contemporary evidence, reflecting the recent development and clinical application of light-based therapies for myopia control. The methodological quality of the included studies was assessed using the Downs and Black checklist, which is a validated tool for assessing both randomized and non-randomized studies. Methodological quality was assessed using a modified 10-item version of the Downs and Black checklist [20], adapted for the heterogeneity of the studies included in this review. The ten items were selected because they apply across all study designs included in the review, as presented in Table 1. Each item was scored 1 (criterion met) or 0 (not met or unclear), giving a total score from 0 to 10. Sources scoring 8–10 were classified as high quality, 5–7 as moderate quality, and 0–4 as low quality, as shown in Table 1. The present systematic review was conducted to assess the original studies on the efficacy and safety of the use of LLRL and short-wavelength therapies in controlling and preventing myopia in children.

### 2.2. Search Policy and Selection Criteria

The search strategy of this systematic review used Boolean operators (AND/OR) to combine relevant keywords and achieve precise retrieval of studies. A comprehensive search of major electronic databases was performed to identify eligible studies published from 2013 to 2026, providing a wide range of evidence within the specified period. The search strategy used the following Medical Subject Headings (MeSH) terms and relevant keywords, combined using Boolean operators (AND/OR) to optimize the retrieval of related studies: (“low-level red light therapy” OR “short-wavelength light therapy”) AND myopia AND children AND (control OR prevention) AND (effectiveness OR efficacy OR safety). This structured approach was applied to ensure comprehensive coverage of studies on the efficacy and safety of LLRL and short-wavelength therapies for myopia prevention and control.

### 2.3. Eligibility and Inclusion Criteria

The present systematic review included peer-reviewed articles published in English that assessed the effectiveness and/or safety of LLRL or short-wavelength light therapy for myopia prevention or control. Eligible study designs included randomized controlled trials (RCTs), non-randomized trials, prospective and retrospective cohort studies, and other comparative observational studies. No restrictions were placed on the comparator, including single-vision spectacles, atropine, orthokeratology, other myopia control interventions, or placebo treatment. The included studies were required to report at least one efficacy outcome (e.g., spherical equivalent refraction, axial length, or myopia incidence) and/or safety outcome. To provide a comprehensive assessment of safety, case reports, case series, and dedicated safety studies reporting adverse events associated with LLRL or short-wavelength light therapy were also eligible. However, studies were excluded if they did not involve pediatric participants, did not evaluate LLRL or short-wavelength light therapy for myopia control or prevention, or lacked sufficient data for analysis. Conference abstracts, editorials, meeting proceedings, reviews, commentaries, letters without original data, animal studies, and laboratory-based investigations were excluded. These criteria are designed to ensure the methodological rigor and reliability of this systematic review.

### 2.4. Data Extraction Process

Reviewers independently screened the titles and abstracts of all identified articles according to a standardized data extraction form. This form is designed to ensure consistency and accuracy in recording important study information. Data extracted included first author name, year of publication, country of study, and characteristics of participants (mean age and sample size, severity of myopia, type of intervention, duration of follow-up, and primary outcome measures of effectiveness and safety). This systematic approach provided a comprehensive and unbiased synthesis of the evidence. In this review, the effectiveness outcomes for the intervention were changes in myopia progression, axial length (AL), spherical equivalent refraction (SER), and choroidal thickness (CT). Safety outcomes included markers of retinal integrity and any evidence of ocular damage following the intervention. These measures were chosen to provide a comprehensive assessment of therapeutic efficacy and potential risks of LLRL and short-wavelength light therapies. To maintain consistency and transparency throughout the entire review process, predetermined standards and protocols were designed to direct the selection, evaluation, and extraction of information from qualified studies. Where there was disagreement between reviewers, formal processes were followed to encourage open dialogue and objective resolution. Disagreements on study selection, data extraction, or quality assessment of the studies were resolved by discussion, according to predefined, evidence-based criteria rather than subjective judgment. In the absence of a consensus, a third-party expert in the field was consulted to provide independent and neutral adjudication. The measures are meant to improve the reliability, accuracy, and credibility of the systematic review.

### 2.5. Methodological Quality Assessment

The methodological quality of each study included in this systematic review was assessed using the Downs and Black checklist [20], a validated and widely accepted tool for the assessment of randomized and non-randomized studies, as presented in Table 1. The reason for choosing this checklist is that it covers a broad range of domains: quality of reporting, external validity, internal validity, and statistical power. This flexibility is especially useful for systematic reviews with different study designs, allowing a thorough and robust assessment of methodological quality. To maintain objectivity and consistency, a methodological assessment of all eligible studies in this systematic review was conducted by two independent reviewers. Discrepancies or disagreements in the evaluation process were discussed according to the predefined criteria. If consensus could not be reached, a third reviewer provided an independent opinion to help resolve the matter. This approach is designed to ensure methodological rigor and to minimize bias across the review.

## 3. Results

### 3.1. Studies Characteristics

**Figure 1:**
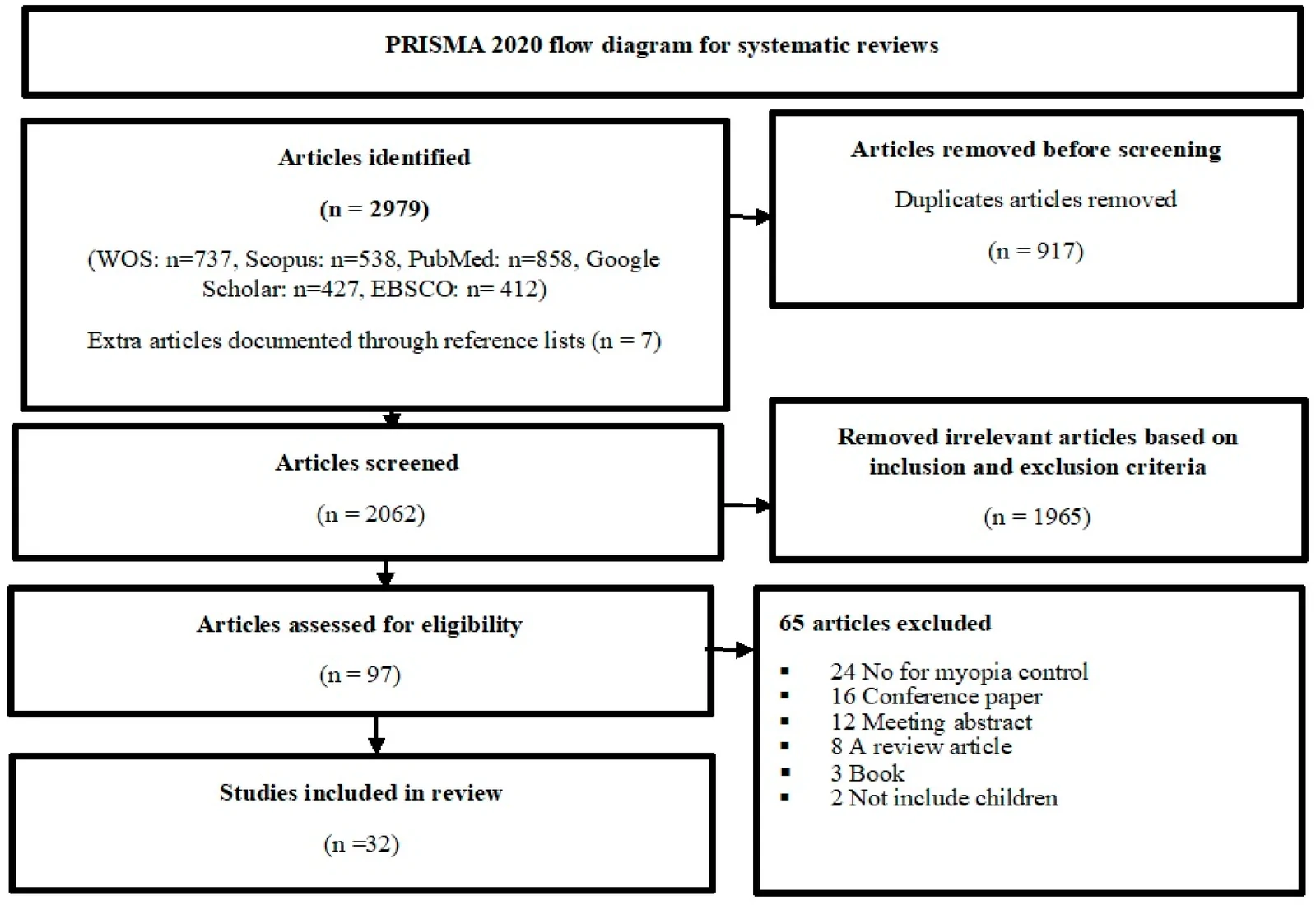
PRISMA 2020 flow diagram for systematic review.

A total of 2979 articles were identified (Figure 1). After de-duplication, 2062 titles were screened. Of these, 1965 were excluded after abstract review for not meeting the predefined inclusion criteria for the study. Additionally, 65 study titles were excluded as they were not related to myopia control or prevention; did not include children; were conference abstracts, review articles, or book chapters; or lacked sufficient extractable data. The details of the study selection process are shown in Figure 1. Finally, 32 quality-assessed studies [11,12,21,22,23,24,25,26,27,28,29,30,31,32,33,34,35,36,37,38,39,40,41,42,43,44,45,46,47,48,49,50,51,52] from six countries were included in this systematic review and are summarized in Table 2. These studies, published between 2013 and 2026, included a total of 5782 children with a mean age of 9.98 ± 1.87 years. The included studies assessed the main outcomes of effectiveness, such as changes in myopia, AL, SER, and CT. Moreover, safety outcomes were assessed by determining retinal integrity and reporting any possible ocular adverse effects of LLRL and short-wavelength light therapies for myopia control and prevention.

Table 3 represents the mapping of the included LLRL studies, which identified several study families with potential participant overlap. The largest cluster, Family A, comprised five publications derived from the same parent cohort, including post hoc, follow-up, and subgroup analyses. Additional study families (B–F) included two to three related publications each, with possible or uncertain cohort overlap based on shared authorship, research networks, or similar study designs. In contrast, eight studies appeared to represent independent cohorts with no obvious connection to the major LLRL research networks.

### 3.2. Efficacy and Safety of LLRL Therapy

#### 3.2.1. Effectiveness

The 26 studies are summarized in Table 4. LLRL therapy demonstrated strong effectiveness and promising safety for controlling and preventing myopia in children. Notably, all these studies were conducted in China. In the included studies, Jiang et al. 2022 [11], Tian et al. 2022 [12], Wang et al. 2023 [21], Cao et al. 2024 [22] and Chen et al. 2023 [23] reported significantly reduced AL elongation and SER progression in children treated with LLRL therapy compared to controls. Moreover, studies [21,22,23,24,25,26,27,28,29,30,31,32,33,34,35,36,37,38,39,40,41,42,43,44] also reported AL shortening and stabilized refractive outcomes over the treatment duration ranging from 6 months to 3 years, as summarized in Table 3 and Table 5. Additionally, a significant increase in macular choroidal thickness was observed in several studies conducted by Chen et al. 2023 [23], Zhou et al. 2022 [32] and Xiong et al. 2023 [35], suggesting a structural mechanism for treatment response. In addition, LLRL was also effective in delaying or preventing the onset of myopia in premyopic children compared with 0.01% atropine in some comparative RCTs, including the studies by Chen et al. 2022 [34] and Zhang et al. 2025 [39].

#### 3.2.2. Safety

Regarding the safety outcomes of studies evaluating LLRL therapy [11,12,21,22,23,24,25,26,27,28,29,30,31,32,33,34,35,36,37,38,39,40,41,42,43,44,45,46,47,48,49,50,51,52], some studies reported retinal structural and functional changes. Transient manifestations including OCT hyperreflectivity, color perception changes, dry eye symptoms, or conjunctivitis were reported among participants by Zhu et al. 2024 [36], Zhang et al. 2025 [39], Xu et al. 2024 [38], Zhang et al. 2025 [41], Chen et al. 2026 [42], and Liao et al. 2025 [44]. Overall, the evidence summarized in Table 4 and Table 6 suggests potential safety concerns associated with LLRL therapy, highlighting the need for further evaluation of its long-term safety despite its promise for myopia control in children.

### 3.3. Efficacy and Safety of Short-Wavelength Light Therapy

#### 3.3.1. Effectiveness

Evidence from clinical, experimental, and observational studies suggests that exposure to short-wavelength light may influence the development and progression of myopia [45,46,47,48,49,50,51,52], as presented in Table 5 and Table 6. The most substantial longitudinal evidence comes from studies [45,46] examining VL-transmitting eyeglasses. Mori et al. 2021 [45] reported that children wearing VL-transmitting lenses had significantly less AL elongation compared with placebo lenses in a two-year RCT, demonstrating a 21.4% reduction in AL growth. Similarly, Torii et al. reported that 3 h of daily ultraviolet 310 nm exposure caused significant short-term suppression of AL elongation, increased choroidal thickness, and changes in SER, supporting the potential role for VL in regulating ocular growth [46]. Short-term experimental studies investigating blue and cyan light wavelengths demonstrated wavelength-dependent biological effects. Blue light exposure inhibited AL elongation, whereas red and green light exposure promoted AL elongation [49]. Cyan light exposure was associated with reduced AL and increased CT, with stronger effects observed in myopic eyes [51]. Other studies suggested a transient increase in CT with blue light stimulation of melanopsin-expressing pathways and modulation of defocus-induced choroidal responses with blue light filtering [52]. However, very short blue light stimulation directed toward the optic disk did not induce significant choroidal changes, highlighting the importance of adequate exposure duration and retinal targeting [48].

#### 3.3.2. Safety

Across all studies reported in Table 5 and Table 6 [45,46,47,48,49,50,51,52], there were no ocular or systemic adverse events with controlled VL, blue, or cyan light exposure. Children tolerated long-term VL exposure via transmitting eyeglasses over a two-year period with no observed retinal, anterior segment or visual complications [45]. Safety concerns have not been reported in observational studies of natural VL exposure among stable myopes [47]. Moreover, exposure to blue and cyan light, ranging from short (1 min) duration targeted stimulation to longer sessions (1–2 h), including light-emitting glasses and blue light modulated defocus models, have consistently shown no ocular symptoms or structural damage in children and young adults [48,49,50,51,52].

## 4. Discussion

This systematic review summarizes the recent evidence regarding the efficacy and safety of LLRL and short-wavelength light therapies for the control and prevention of childhood myopia. LLRL therapy is an emerging and promising non-invasive therapeutic modality for the control and prevention of childhood myopia, as demonstrated in the studies [11,12,21,22,23,24,25,26,27,28,29,30,31,32,33,34,35,36,37,38,39,40,41,42,43,44] reviewed here and summarized in Table 2, Table 3 and Table 6. We reviewed 34 studies that included 5837 children to evaluate the efficacy and safety of LLRL and short-wavelength light interventions for myopia control, and most of the studies were RCTs with high methodological quality, thereby improving the reliability of the present systematic review. Interestingly, most of the included studies were conducted in China, which reflects the high prevalence of pediatric myopia in East Asia [2] and the early implementation of LLRL intervention for myopia prevention and control in this area.

### 4.1. Effectiveness of LLRL Therapy on Myopia Development

Most studies demonstrated a clinically significant reduction in myopia development, evidenced by slower AL elongation and reduced SER progression. In large RCTs, LLRL therapy had significantly lower axial elongation compared with controls wearing single-vision spectacles or receiving placebo treatment [11,12,21,22]. In post hoc analysis, LLRL therapy was associated with reversal of axial elongation in one-quarter of participants, indicating a potential regressive effect on AL in a subgroup of individuals [21]. Furthermore, in a retrospective cohort of 434 myopic children, more than one-quarter revealed AL shortening greater than 0.05 mm following LLRL therapy, a phenomenon rarely reported with other myopia control methods such as low-dose atropine or special design optical interventions [33]. The effect size was large, with reductions in axial elongation of approximately 0.20–0.30 mm over one year in the RCTs by Jiang et al. [11] and Tian et al. [12] versus controls, with satisfactory changes in SER. These findings were confirmed in subsequent studies with longer follow-up periods, with two- and three-year data demonstrating sustained efficacy with only a modest rebound after treatment discontinuation [30,39,42]. Based on these results, LLRL therapy was effective for a wide range of baseline myopia severities, from premyopia to high myopia [24,26,38].

Chen et al. [34] reported significantly less AL elongation and slower SER progression with LLRL compared with 0.01% atropine over 12 months, whereas Zhang et al. [39] observed higher proportions of SER regression and AL shortening among children receiving the LLRL group over two years. The LLRL therapy offers some benefits over the existing methods of myopia control, including ones attributable to its non-pharmacological and non-invasive nature and good acceptability. In some cases, the LLRL therapy demonstrated a reversal of axial elongation, a finding rarely seen with low-dose atropine or optical interventions for myopia control. Moreover, LLRL therapy seems to be effective across a wide range of baseline myopia severities and may achieve treatment effects comparable to or potentially greater than those observed with low-dose atropine, without drug-related side effects such as photophobia or near blur. LLRL therapy may be limited by availability, higher cost and the need for strict adherence to treatment protocols. Furthermore, unanswered questions remain regarding long-term safety, optimal treatment protocols and durability of effects after discontinuation. Overall, the current evidence suggests that LLRL may represent a useful non-pharmacological option or adjunctive strategy for myopia control and prevention. Differences in ethnicity, geographic location, environmental exposures, myopia progression patterns, treatment adherence, and clinical practice may influence treatment outcomes. As all included LLRL studies were conducted in China, further multinational studies are needed to confirm the generalizability of these findings across diverse populations and clinical settings.

The precise biological mechanisms underlying the myopia control effect of LLRL remain largely unknown; however, several studies have provided insights into potential mechanisms. Several studies reported increased choroidal thickness after LLRL therapy, especially in the subfoveal region, along with the improvement of choroidal vascular parameters [23,26,32,35,40]. Previous studies have shown that choroidal thickening is associated with reduced AL elongation and may represent a protective factor for myopia progression [26,35,40]. The potential mechanistic link between choroidal structural changes and treatment efficacy may be explained by the following: LLRL treatment has been reported to improve choroidal blood perfusion, choroidal vascularity index, and choriocapillaris luminal area [53]. These modifications in vascular parameters were significantly associated with a slower rate of AL elongation and refractive progression [53]. Transient increases in retinal thickness have also been reported after short-term LLRL exposure, possibly reflecting alterations in retinal metabolism or signaling pathways [28]. Experimental data suggest that red light exposure may modulate mitochondrial activity, retinal oxygenation, and scleral remodeling, which may collectively contribute to reduced axial elongation. In some studies, the observed dose–response relationship, in which different power outputs have been associated with different degrees of myopia control, supports a biologically driven effect rather than a placebo response [31,32]. The retina and optic nerve are rich in mitochondria, consistent with their high energy demands for visual signal transduction and intracellular transport. Red light irradiation has been proposed as a potential photobiomodulation strategy for myopia control because mitochondrial chromophores, particularly cytochrome c oxidase, absorb red wavelengths [54].

Several included studies [11,12,21,22,23,24,25,26,27,28,29,30,31,32,33,34,35,36,37,38,39,40,41,42,43,44] reported LLRL-associated changes in CT, changes in retinal structure, and reduction in axial elongation; these findings primarily reflect associations rather than definitive causal mechanisms. Proposed pathways, including photobiomodulation, mitochondrial activation, improved choroidal perfusion, and modulation of scleral remodeling, are supported by indirect clinical observations and preclinical evidence. Direct mechanistic evidence associated between these biological changes to myopia control remains limited, with few studies examining temporal relationships, dose–response effects, mediation analyses, or pathway-specific molecular and cellular outcomes. Therefore, the mechanistic explanations for LLRL should be considered biologically possible hypotheses rather than established mechanisms. Future research should include longitudinal mechanistic investigations, molecular biomarkers, and pathway-targeted assessments to explain the causal pathways underlying the observed therapeutic effects of LLRL in myopia control.

### 4.2. Safety Considerations of LLRL Therapy in Myopia Management

Adverse events were reported in some of the included studies of LLRL therapy [11,12,21,22]. Long-term cohort studies [42,43] of more than two to three years’ duration reported retinal structural changes associated with LLRL therapy. Furthermore, some studies have reported retinal or visual effects associated with LLRL therapy, including transient increases in photoreceptor reflectance, altered color perception, dry eye symptoms, conjunctivitis, transient dome-shaped hyperreflectivity between the retinal pigment epithelium and the foveal ellipsoid zone, cystoid changes within the ganglion cell layer, and reductions in paracentral cone density [36,39,41,44]. Although the emerging evidence is promising, information regarding the long-term safety of LLRL therapy has only recently become available and remains limited. A multicenter cohort study with a follow-up period of more than three years reported satisfactory myopia control, defined as an annual axial elongation of ≤0.10 mm, in more than 70% of treated children with stable visual acuity and no evidence of duration-dependent retinal toxicity [42,43]. These ocular surface and retinal changes highlight the need for ongoing ophthalmic monitoring, particularly with longer treatment durations and higher laser power settings. The heterogeneity in reported safety outcomes further emphasizes the need for standardized safety protocols and long-term investigation. This evidence highlights the importance of routine OCT examination, especially for long-term therapy and in younger children, and supports the overall safety of LLRL therapy with appropriate supervision. The results in Table 2, Table 4 and Table 6 indicate that LLRL therapy is an effective option for controlling myopia progression in children. Its non-invasive nature, good acceptability, and high compliance rates make it attractive for pediatric populations. Future multicenter, worldwide RCTs with standardized protocols are needed to confirm its effectiveness in different populations and to determine optimal treatment protocols. The overall safety profile of LLRL remains a concern, as reported retinal structural and functional changes warrant cautious interpretation and further long-term safety evaluation. In addition, further mechanistic studies and rigorous long-term safety assessments will be required before LLRL therapy can be widely recommended as a first-line intervention in global myopia control guidelines.

A notable gap in the current evidence base is the lack of assessment of potential systemic and neuroendocrine effects associated with repeated LLRL exposure. None of the included studies assessed endocrine biomarkers, circadian rhythm regulation, melatonin secretion, sleep parameters, or other indicators of systemic physiological function. Even though available clinical evidence has not identified systemic adverse events, experimental photobiological studies suggest that light exposure may influence neuroendocrine signaling pathways under certain conditions. Consequently, the long-term systemic implications of LLRL exposure during childhood remain insufficiently understood. Future RCTs should include comprehensive systemic safety assessments, including circadian, endocrine, sleep-related, and developmental assessments, to establish a more complete understanding of the long-term safety profile of LLRL therapy in pediatric populations. An important evidence gap remains regarding the potential endocrine, sleep, circadian, and developmental effects of LLRL therapy, because none of the included studies assessed these outcomes. The absence of reported systemic adverse events therefore cannot be taken as evidence of systemic safety, and dedicated long-term studies are needed to evaluate these outcomes.

### 4.3. Effectiveness of Short-Wavelength Light Therapy on Myopia Development

Table 5 and Table 6 summarize the studies [45,46,47,48,49,50,51,52] that demonstrate the increasing interest in short-wavelength visible light therapies as non-pharmacological strategies for myopia control and prevention. The reviewed studies indicate that specific light ranges, particularly violet (360–400 nm), blue (450–470 nm) and cyan (500–510 nm), may influence ocular growth through modulation of AL elongation and choroidal thickness with an overall favorable preliminary safety profile. These investigations included RCTs and prospective human studies conducted in East Asia and Australia, regions heavily affected by the global myopia epidemic. The strongest clinical evidence supporting VL use in myopia control and prevention is from RCTs in children, as shown in Table 2, Table 5 and Table 6, particularly those conducted in Japan by Mori et al. [45] and Torii et al. [46]. These RCTs were of high methodological quality and included children aged 6–12 years, a population at high risk of rapid myopia progression. However, most adult studies were prospective and focused on short-term physiological outcomes such as changes in AL and choroidal thickness, rather than long-term refractive progression [47,48,50,51]. Some studies [47,48,49,50,51,52] were limited by small sample sizes; however, the use of methodologically consistent approaches (e.g., objective biometric outcomes, controlled wavelength exposure, and repeated-measures designs) strengthens the internal validity of their findings. However, the substantial heterogeneity in wavelengths used, exposure times, and outcome measures limits quantitative comparisons across studies and reduces the strength of evidence synthesis.

Among the interventions reviewed, VL-transmitting spectacles demonstrated the most clinically meaningful and sustained effects. Mori et al. [45] reported a 21.4% reduction in AL elongation over two years among children wearing VL lenses compared with placebo lenses, with no reported adverse events. Torii et al. [46] also found significantly less AL elongation with controlled ultraviolet 310 nm exposure, with favorable changes in CT and SER over shorter follow-up periods. These clinical findings support the hypothesis that VL exposure may reproduce key protective components of outdoor daylight that are reduced in indoor settings due to the filtration of shorter wavelengths by modern windows and conventional spectacles, which block wavelengths below 400 nm. This kind of exposure to VL may stimulate the retinal photoreceptors and thereby contribute to regulatory pathways involved in ocular growth modulation [45,46]. VL is suggested to operate through activation of neuropsin (OPN5), a non-visual photopigment expressed in retinal and choroidal tissues, which may contribute to choroidal thickness regulation and scleral remodeling. A recent study [55] suggests that VL exposure may synergize with dopamine-mediated signaling pathways. Both pathways have been independently associated with inhibition of excessive AL elongation and reduced myopia progression, providing evidence for a biological pathway linking spectral light exposure to ocular growth regulation.

Some studies [48,49,50,51,52] using short-term stimulation protocols have investigated the effects of blue and cyan light exposure on ocular structures, and their results are summarized in Table 4. These studies looked at short-term structural and functional changes and not long-term changes in refractive development or AL growth, making direct comparisons with myopia control interventions difficult. Thakur et al. [49] found that blue light (460 nm) induced a transient decrease in AL, whereas red and green light were associated with AL elongation. Likewise, Chakraborty et al. [51] reported that exposure to cyan light (507 nm) reduced AL and increased choroidal thickness in children and young adults, with a greater effect observed in the myopic eyes. These findings support the hypothesis that short-wavelength visible light may induce rapid changes in ocular biometrics through signaling pathways related to retinal defocus detection and choroidal blood flow control.

The short-wavelength hypothesis was also supported by Hoseini et al. [50], who found increased subfoveal choroidal thickness after a short period of blue light stimulation, which suggests that the melanopsin-mediated pathways may be involved in ocular growth regulation. Melanopsin is a photosensitive protein expressed in a subset of retinal ganglion cells. It is predominantly responsive to short-wavelength (blue) light and plays a key role in regulating non-visual functions, including circadian rhythm entrainment, pupillary light reflex, and hormonal modulation. These effects occur independently of the rod and cone pathways responsible for visual function [56]. A recent study revealed that blue light stimulation of the blind spot induces choroidal thickening and AL shortening in children independent of refractive error and time of day. Similar effects with morning and afternoon stimulation suggest minimal diurnal variation. Conversely, only minor choroidal modifications were seen in myopic adults, suggesting that children may exhibit greater age-related responsiveness to blue light stimulation [51]. Furthermore, Hoseini et al. [50] showed that blue light stimulation targeting melanopsin at the blind spot increases CT in myopic subjects. Some studies have reported favorable ocular biometric changes [49,50,51,52]. However, findings from blue light investigations have not been entirely consistent. Mehler et al. [48] did not observe significant changes in choroidal thickness after short-duration blue light stimulation of the optic disk, suggesting that exposure duration, retinal location, and stimulation protocol may be critical determinants of efficacy. These differences suggest that short-term or isolated stimulation may be insufficient to induce sustained retinal dopaminergic activation relevant to myopia prevention and control. Wu et al. [52] showed that blue light filtering can modify the myopia control effects associated with defocus, suggesting that optical defocus and the spectral light composition may interact. Taken together, these findings suggest that light-based interventions may be most effective when carefully optimized and potentially combined with optical or behavioral strategies. Overall, the evidence summarized in Table 5 suggests that spectrum-specific light therapy may represent a promising adjunctive or alternative approach for myopia control and prevention, particularly VL interventions in children. Light-based strategies may offer a non-invasive option with high acceptability and few side effects, compared to pharmacological treatments such as atropine. Long-term RCTs are needed to establish standardized treatment protocols and to explore combined approaches such as light therapy combined with optical defocus or behavioral interventions to optimize myopia control efficacy.

### 4.4. Safety Considerations of Short-Wavelength Light Therapy in Myopia Management

Importantly, none of the studies reviewed in Table 5 and Table 6 [45,46,47,48,49,50,51,52] reported any significant adverse ocular effects following repetitive or daily exposure to violet, blue, or cyan light. Overall, the findings suggest that short-wavelength light interventions applied within defined limits of intensity and exposure duration appear to be clinically safe in pediatric populations. However, most included studies had short follow-up periods and small sample sizes. Therefore, the long-term ocular safety of these interventions in children with developing visual systems remains incompletely characterized and requires further investigation by well-designed longitudinal RCTs with larger sample sizes. Violet light (380–450 nm) is a high-energy visible light that may induce oxidative stress through the generation of reactive oxygen species, potentially increasing the risk of photochemical retinal damage. Prolonged exposure to short-wavelength light has also been associated with lens and retinal damage. Furthermore, isolated reports of retinal damage following repeated RLRL therapy highlight the need for continued safety monitoring. Therefore, while light-based therapies show promise for myopia control, their long-term ocular safety warrants further investigation.

The findings of this review should be interpreted in the context of the substantial heterogeneity between LLRL therapy and shorter-wavelength light interventions. These methods differ in wavelength, delivery techniques, exposure protocols, proposed biological mechanisms, and stage of clinical development. Therefore, rather than representing a single therapeutic category, they should be considered distinct evidence streams. While both aim to reduce myopia progression, direct comparisons of their efficacy and safety should be made with caution, and further studies are needed to clarify their respective mechanisms and clinical roles. Furthermore, SER outcomes should be interpreted with caution when the use of cycloplegia is unclear, as accommodative bias may influence refractive measurements. In such cases, axial length represents a more objective and reliable indicator of treatment efficacy.

The axial shortening reported in some LLRL studies should be interpreted with caution. Despite the fact that this finding may indicate a significant treatment effect, simultaneous increases in choroidal thickness suggest that changes in ocular structures may contribute to the observed reduction in AL. Whether axial shortening represents sustained structural shortening of the eye remains uncertain. Furthermore, evidence regarding post-treatment rebound remains limited, and the long-term stability of these changes has not been fully established. Further longitudinal studies with extended follow-up are required to clarify the mechanisms underlying axial shortening and the potential for rebound after treatment cessation.

There were some limitations of the present systematic review which need to be acknowledged. First, all included studies assessing LLRL therapy were conducted in China, with many originating from closely related research groups. While this reflects the current evidence base, such geographic and institutional clustering may increase the risk of publication bias and limit the external validity of the findings. Consequently, the generalizability of these results to other ethnic populations, geographic regions, and healthcare settings remains uncertain and should be confirmed through studies conducted by independent research teams in diverse populations. Second, there was a substantial heterogeneity in the LLRL therapy and short-wavelength light therapy protocols, including differences in light power, wavelength, exposure time, type of device, follow-up times, and outcome measures, thereby limiting direct comparison between studies. Third, the sample sizes were small, particularly in studies investigating short-wavelength light therapy, which may have reduced statistical power and limited the generalizability of the findings. Moreover, many investigations focused on short-term anatomical changes, such as retinal and choroidal alterations, rather than long-term effects on myopia progression. While neuroretinal or retinal safety outcomes beyond three to five years remain insufficiently characterized, particularly among children, long-term evidence is still emerging. A substantial proportion of the included evidence was derived from observational, non-randomized, and small-scale studies, which are inherently prone to selection bias, residual confounding, and other methodological limitations compared with RCTs. Furthermore, potential confounding factors, such as outdoor activity, near-work exposure, and concurrent myopia control interventions, were not uniformly controlled across studies. The current evidence is the inconsistent reporting of light-exposure parameters; notably, a reported wavelength of 310 nm is inconsistent with the commonly accepted VL range (360–400 nm), highlighting the need for standardized and accurate characterization of light-based interventions. This review assessed methodological quality with a modified Downs and Black checklist rather than a formal risk-of-bias tool, which limits how confidently potential sources of bias can be evaluated. Finally, few studies have directly compared LLRL or short-wavelength light therapies with combination treatment approaches that may more closely reflect current clinical practice.

## 5. Conclusions

This systematic review highlights light-based therapies, particularly LLRL and short-wavelength light interventions, as promising non-pharmacological approaches for myopia control and prevention in children. The findings indicate that LLRL therapy is effective and consistently associated with reductions in AL elongation, changes in CT, and slower SER progression across multiple high-quality RCTs. Some studies have reported ocular surface and retinal changes. These include changes in photoreceptor characteristics, altered color perception, dry eye symptoms or conjunctivitis, dome-shaped foveal hyperreflectivity, cystic changes in the ganglion cell layer, and reduced paracentral cone density. These findings highlight the importance of regular retinal monitoring, particularly during long-term treatment in children. Short-wavelength light-based strategies, particularly VL-transmitting lenses, have also demonstrated clinically meaningful benefits, potentially through mechanisms involving choroidal thickening and retinal photoreceptor-mediated signal pathways that may replicate protective aspects of outdoor light exposure. In addition, the reviewed studies on blue and cyan light therapy suggest wavelength-dependent effects on ocular biometry, particularly in children; however, evidence supporting sustained refractive outcomes remains limited. These positive findings are balanced by important limitations, including the geographical concentration of the available studies, substantial heterogeneity of light parameters and treatment protocols, relatively small sample sizes (particularly for short-wavelength light therapy), and short follow-up periods. Future longitudinal, multicenter RCTs with standardized protocols are required to help clarify optimal treatment parameters and define the role of light-based therapies within comprehensive myopia management strategies.

## Figures and Tables

**Table 1:** Quality assessment of included studies in the systematic review using the Downs and Black checklist.

Author and Year	10-Item for Assessment										Quality Score
	Objective	Outcomes	Characteristics	Sample Size	Findings	*p*-Value	Data Cleaning	Variability	Statistical Tests	Validity	
Jiang et al. 2022 [11]	1	1	1	1	1	1	1	1	1	1	10
Tian et al. 2022 [12]	1	1	1	1	1	1	1	1	1	1	10
Wang et al. 2023 [21]	1	1	1	1	1	1	1	1	1	1	10
Cao et al. 2024 [22]	1	1	1	1	1	1	1	1	1	1	10
Chen et al. 2023 [23]	1	1	0	1	1	1	1	1	1	1	9
He et al. 2023 [24]	1	1	1	1	1	1	1	1	1	1	10
Dong et al. 2023 [25]	1	1	0	1	1	1	1	1	1	1	9
Liu et al. 2024 [26]	1	1	1	1	1	1	1	1	1	1	10
Lin et al. 2023 [27]	1	1	1	1	1	1	1	1	1	1	10
Yang et al. 2023 [28]	1	1	0	0	1	1	1	1	1	1	8
Wang et al. 2023 [29]	1	1	1	0	1	1	1	1	1	1	9
Xiong et al. 2022 [30]	1	1	1	1	1	1	1	1	1	1	10
Zhou et al. 2024 [31]	1	1	1	1	1	1	1	1	1	1	10
Zhou et al. 2022 [32]	1	1	0	1	0	1	1	1	1	1	8
Wang et al. 2023 [33]	1	1	1	1	1	1	1	1	1	1	10
Chen et al. 2022 [34]	1	1	1	1	1	1	1	1	1	1	10
Xiong et al. 2023 [35]	1	1	1	1	1	1	1	1	1	1	10
Zhu et al. 2024 [36]	1	1	1	1	1	1	1	1	1	1	10
Yang et al. 2025 [37]	1	1	0	1	1	1	1	1	1	1	9
Xu et al. 2024 [38]	1	1	1	1	1	1	1	1	1	1	10
Zhang et al. 2025 [39]	1	1	1	1	0	1	1	1	1	0	8
Xiang et al. 2025 [40]	1	1	1	1	1	1	1	1	1	1	10
Zhang et al. 2025 [41]	1	1	1	1	1	1	1	1	1	1	10
Chen et al. 2026 [42]	1	1	0	1	1	1	1	1	1	1	9
Lili et al. 2025 [43]	1	1	0	1	1	1	1	1	1	1	9
Liao et al. 2025 [44]	1	1	1	1	0	1	1	1	1	0	8
Mori et al. 2021 [45]	1	1	1	1	1	1	1	1	1	1	10
Torii et al. 2022 [46]	1	1	1	1	1	1	1	1	1	1	10
Mehler et al. 2025 [47]	1	1	1	0	1	1	1	1	1	1	9
Thakur et al. 2021 [48]	1	1	1	0	1	1	1	1	1	1	9
Chakraborty et al. 2024 [49]	1	1	1	0	1	1	1	1	1	1	9
Wu et al. 2023 [50]	1	1	1	0	1	1	1	1	1	1	9

**Table 2:** Characteristics of included studies assessing the effectiveness and safety of LLRL and short-wavelength light therapy for myopia control and prevention.

Author and Year	Country	Age Group (Years)	Age (Mean (SD)	Study Design	Sample Size	IRB or Trial Registry IDs
Jiang et al. 2022 [11]	China	8–13	8.6 ± 2.6	RCT	264	NCT04073238
Tian et al. 2022 [12]	China	6–12	9.66 ± 1.65	RCT	224	TRECKY2021-239
Wang et al. 2023 [21]	China	8–13	8.6 ± 2.6	RCT	264	2019KYPH093
Cao et al. 2024 [22]	China	6–12	9.1 ± 2.0	RCT	336	ChiCTR2200058963
Chen et al. 2023 [23]	China	6–13	-	RCT	102	ChiCTR2100045250
He et al. 2023 [24]	China	6–11	8.3 ± 1.1	RCT	287	NCT04825769
Dong et al. 2023 [25]	China	7–12	-	RCT	112	ChiCTR2100052322
Liu et al. 2024 [26]	China	7–12	8.98 ± 1.31	RCT	94	ChiCTR2200062028.
Lin et al. 2023 [27]	China	6–18	8.54 ± 2.82	RCT	164	ChiCTR2000032172
Yang et al. 2023 [28]	China	6–12	-	Prospective	25	2021106-1
Wang et al. 2023 [29]	China	6–12	-	Comparison	51	2021KY-14
Xiong et al. 2022 [30]	China	8–13	10.46 ± 1.33	RCT	199	NCT04073238
Zhou et al. 2024 [31]	China	6–15	-	RCT	200	ChiCTR2200058615
Zhou et al. 2022 [32]	China	-	9.19 ± 2.40	Retrospective	105	Not available
Wang et al. 2023 [33]	China	3–17	9.7 ± 2.6	Prospective	434	2020KYPJ107
Chen et al. 2022 [34]	China	7–15	9.78 ± 1.58	RCT	62	ChiCRT2100045834
Xiong et al. 2023 [35]	China	8–13	-	RCT	120	NCT04073238
Zhu et al. 2024 [36]	China	6–14	8.96 ± 2.19	Prospective	108	ChiCTR2100043085
Yang et al. 2025 [37]	China	8–10	-	RCT	116	NCT06683287
Xu et al. 2024 [38]	China	6–16	10.40 ± 2.4	RCT	192	NCT05184621
Zhang et al. 2025 [39]	China	7–16	11.6 ± 1.9	Retrospective	122	2004,001
Xiang et al. 2025 [40]	China	6–10	8.27± 1.10	RCT	463	2021]022
Zhang et al. 2025 [41]	China	7–12	8.43 ± 1.53	RCT	86	ChiCTR2400090938
Chen et al. 2026 [42]	China	7–18	-	Cohort	362	NCT05871840
Lili et al. 2025 [43]	China	6–18	-	Prospective	896	Not available
Liao et al. 2025 [44]	China	5–14	10.3 ± 1.9	Retrospective	99	Not available
Mori et al. 2021 [45]	Japan	6–12	9.5 ± 1.5	RCT	113	RCTs032180418
Torii et al. 2022 [46]	Japan	6–12	9.8 ± 1.7	RCT	43	UMIN 000036453
Mehler et al. 2025 [47]	Germany	-	-	Prospective	46	22-1023
Thakur et al. 2021 [48]	India	-	-	Prospective	25	LEC 05-19-256
Chakraborty et al. 2024 [49]	Australia		12.52 ± 2.40	Prospective	44	HREC, ID: 2877
Wu et al. 2023 [50]	China	-	-	RCT	24	NCT06449976
All			9.98 ± 1.87		5782	

Randomized controlled trial = RCT.

**Table 3:** Study-family and cohort mapping of included LLRL Studies.

Study Family/Parent Cohort	Related Publications Included in Review	Relationship Between Publications	Potential Overlap
**Family (A)**	Jiang et al. 2022 [11]; Wang et al. 2023 [21]; Xiong et al. 2022 [30]; Wang et al. 2023 [33]; Xiong et al. 2023 [35]	Wang et al. [21] was a post hoc analysis of the Jiang et al. [11] RCT; Xiong et al. [30] reported the 2-year follow-up results; Wang et al. [33] assessed axial length shortening; and Xiong et al. [35] investigated longitudinal changes in CT.	High possibility of cohort overlap
**Family (B)**	Chen et al. 2023 [23]; Zhou et al. 2024 [31]	Same research network; potential participant overlap.	Possible overlap
**Family (C)**	He et al. 2023 [24]; Liu et al. 2024 [26]; Xiang et al. 2025 [40]	Premyopic studies shared overlapping authors (He, Zhu, Xiang, Wang), with Liu et al. and Xiang et al. likely analyzing different outcomes from related cohorts.	Possible overlap
**Family(D)**	Chen et al. 2022 [34]; Zhang et al. 2025 [39]	Both studies compared LLRL with low-dose atropine and reported similar efficacy outcomes. Their comparable designs suggest they may be part of related clinical research programs, although direct cohort overlap cannot be confirmed.	Uncertain
**Family(E)**	Chen et al. 2026 [42]; Lili Wang et al. 2025 [43]	Both studies reported the long-term real-world effectiveness and safety of LLRL and shared several authors (Chen, Wang, Jiang, and Zeng), suggesting potentially overlapping cohorts. However, the extent of participant overlap remains unclear.	Uncertain
**Family (F)**	Zhu et al. 2024 [36]; Zhang et al. 2025 [41]; Liao et al. 2025 [44]	These studies primarily assessed retinal/OCT safety outcomes and may have recruited participants from established LLRL treatment programs.	Uncertain
**Independent Chinese**	Tian et al. 2022 [12]; Cao et al. 2024 [22]; Dong et al. 2023 [25]; Zhou et al. 2022 [32]; Yang et al. 2023 [28]; Wang et al. 2023 [29]; Yang et al. 2025 [37]; Xu et al. 2024 [38]	No clear evidence from the author lists suggests a direct link to the major He/Zeng/Zhu/Jiang/Wang/Xiong research network.	No obvious overlap

**Table 4:** Studies used LLRL therapy for myopia control and prevention.

Author and Year	Myopia Severity	Details of Intervention	Follow Up (Year)	Outcome Measure	Safety	Effectiveness
Jiang et al. 2022 [11]	Cycloplegic SER: −1.00 to−5.00 D	A desktop LLRL therapy emitting 650 nm light at about 1600 lux. 3 min twice daily, at least 4 h, 5 days a week.	1.00	Slowed progression (0.13 mm AL elongation, −0.20 D SER) compared to plus SVS (0.38 mm, −0.79 D).	No adverse event	Effective and well-tolerated myopia control option for children.
Tian et al. 2022 [12]	Cycloplegic SER: −0.5 to −6.00 D	Head-mounted device emitted 650 nm light. 3 min twice daily, at least 4 h interval.	0.5	AL changed by −0.06 mm with LLRL vs. +0.14 mm in controls and SER by +0.125 D vs. −0.25 D, respectively (*p* < 0.001)	No adverse event	650 nm LLRL significantly slowed myopia development in children aged 6–12.
Wang et al. 2023 [21]	Cycloplegic SER: −1.00 to−5.00 D	LLRL emitting 650 nm light at 1600 lux. 3 min twice daily, at least 4 h, 5 days a week.	1.00	AL shortening >0.05 mm in 21.9% of LLRL vs. 1.4% of controls (*p* < 0.001).	No adverse event	Approximately one-quarter of children showed AL shortening >0.05 mm.
Cao et al. 2024 [22]	Cycloplegic SER: −3.00 to −6.00 D	Head-worn 650 nm, 3 min twice daily, at least 4 h apart.	1.00	SER changed by +0.15 D vs. −0.26 D at 6 months and +0.24 D vs. −0.65 D at 12 months (*p* < *0*.001). AL changed by −0.06 mm vs. +0.13 mm at 6 months and −0.11 mm vs. +0.26 mm at 12 months for the LLRL and control groups, (*p* < *0*.001).	No adverse event	One year of daily 650 nm LLRL slows SER and AL progression.
Chen et al. 2023 [23]	Not reported	A 635 nm red light laser device with a power of 0.35 ± 0.02 mW.	1.00	LLRL group showed minimal progression (AL +0.01 mm, SER +0.05 D) compared with controls (+0.39 mm, −0.64 D; *p*<0.05) and had a greater increase in SFCT (*p* < 0.05).	No adverse event	LLRL effectively helps prevent and control myopia and may also improve accommodative function.
He et al. 2023 [24]	Cycloplegic SER: More than −0.50 D	The head-mounted device emitted 650 nm light. 3 min twice daily.	1.00	Myopia incidence was lower with LLRL (40.8% vs. 61.3%), and the treatment slowed AL elongation (0.30 vs. 0.47 mm) and SER decline (–0.35 vs. –0.76 D).	No adverse event	LLRL therapy is an effective and well-tolerated method for preventing myopia, reducing incidence to 54.1% in premyopic children.
Dong et al. 2023 [25]	Cycloplegic SER: at least −0.50 D	Children used a home desktop red light device for 3 min, twice daily, 4 h between sessions.	0.5	SER changed by 0.06 ± 0.30 D LLRL vs. 0.11 ± 0.33 D control group (*p* = 0.003), and AL increased 0.02 ± 0.11 mm vs. 0.13 ± 0.10 mm, respectively (*p* < 0.001).	No adverse event	Full-power LLRL therapy (100%) significantly slowed myopia progression over 6 months compared with the 10%-power sham device.
Liu et al. 2024 [26]	Cycloplegic SER: −0.50 +0.75 D	A desktop LLRL therapy emitting 650 nm light at about 1600 lux. 3 min twice daily, 4 h apart.	1.00	Foveal CT increased after starting LLRL therapy, peaking at 3 months. Smaller increases were seen at 1, 6, 9, and 12 months, with the lowest rise at 12 months.	No adverse event	Reduced SER progression by 73.8%, slowed AL elongation by 67.9%, and lowered myopia incidence by 45.1% in one year.
Lin et al. 2023 [27]	Not reported	650 nm LLRL device for 3 min per session, twice daily, 4 h, 5 days a week. After each session, relaxed with eyes closed for 2 min.	60.42 days	AL changes among groups: control (0.08 ± 0.40 mm) and intervention (−0.07 ± 0.11 mm), with *p* < 0.001.	No adverse event	LLRL showed a clear benefit in controlling myopia and played an important role in treating severe cases.
Yang et al. 2023 [28]	Cycloplegic SER: 0.83 ± 1.0 D	LLRL therapy eye was connected to the device and exposed to 650 nm red light for 3 min.	60 days	Retinal thickness in LLRL eyes increased significantly 5 min after treatment, rising by 1.70 ± 0.83% (*p* = 0.0389).	No adverse event	3 min of LLRL therapy does not affect choroid; it causes a brief, transient increase in retinal thickness.
Wang et al. 2023 [29]	Cycloplegic SER: −2.9 ± 1.8 D	LLRL for 3 min, twice daily (≥4 h), 650 nm red light device (1200–1800 lux).	3 Months	AL and SER changes were –0.08 mm and +0.23 D in the LLRL group, compared with +0.08 mm and –0.07 D in the control group.	No adverse event	LLRL treatment can slow axial growth, myopia progression, and binocular anisometropia.
Xiong et al. [30]	Cycloplegic SER: −1.00 to −5.00 D	650 nm red light desktop device at home, for 3 min per session, twice daily, 5 days a week.	2.00	Over 2 years, the smallest AL elongation and SER progression occurred in the LLRL group, and the highest in the SVS group.	No adverse event	LLRL safely and effectively slowed myopia over 2 years, with minimal rebound after stopping.
Zhou et al. 2024 [31]	Cycloplegic SER: −0.5 D or more	The device had three 650 nm light settings (0.37 mW, 0.60 mW, and 1.20 mW). Performed twice daily for 3 min, 4 h.	0.5	After 6 months, SER progression was lower in the 0.37 mW (0.01 D), 0.60 mW (0.05 D), and 1.20 mW (0.16 D) groups compared with the control group (0.22 D; *p* < 0.001 for all).	No adverse event	LLRL effectively reduced myopia development at all tested power levels (0.37, 0.60, and 1.20 mW).
Zhou et al. 2022 [32]	Cycloplegic SER: −3.09 ± 1.74 D	LLRL was applied twice daily (3 min per session) using a 0.4 mW, 635 nm device, with at least 4 h between sessions.	9 months	LLRL group showed less SER progression (−2.87 D vs. −3.57 D, *p* < 0.001) and reduced AL change (−0.06 mm vs. 0.26 mm, *p* < 0.001) compared with controls. CT increased by 45.32 μm in the LLRL group (*p* < 0.001).	No adverse event	LLRL therapy slowed myopia progression and reduced AL growth even over short treatment periods.
Wang et al. 2023 [33]	Cycloplegic SER: −3.74± 2.60 D	LLRL therapy provided by a home-use desktop light device that released light at 650 nm.	1.00	AL shortening happened in 26.5% at ≥0.05 mm/year, 17.5% at ≥0.10 mm/year, and 4.6% at ≥0.20 mm/year.	No adverse event	Over a quarter showed AL shortening >0.05 mm after LLRL therapy, with a mean AL change of −0.142 mm/year.
Chen et al. 2022 [34]	Cycloplegic SER: −2.60 ±1.17 D	The device emits 650 ± 10 nm LLRL at 1600 lux. At home for 3 min twice daily, with at least 4 h between sessions.	1.00	After 1 year, AL increased 0.08 mm in the LLRL group vs. 0.33 mm in the 0.01% atropine group (*p* < 0.001). SER changed by −0.03 D with LLRL vs. −0.60 D with atropine (*p* < 0.001).	No adverse event	LLRL was more effective than 0.01% atropine in controlling AL and myopia progression over 12 months.
Xiong et al. 2023 [35]	Cycloplegic SER: 1.00 to 5.00 D	LLRL therapy was provided using desktop light device that emitted red light at 650 nm.	1.00	Macular CT increased to 14.76 µm at 1 month, dropped to 5.29 mm (3 months) and 1.54 mm (6 months), then increased to 9.09 mm at 12 months.	No adverse event	LLRL caused sustained choroidal thickening.
Zhu et al. 2024 [36]	Cycloplegic SER: −0.50 to −6.00 D	The LLRL device emitted at 650 ± 10 nm, with power of 2 mW and 0.29 Mw.	1.00	AL was shorter and SER was lower in the LLRL group than in the control group	Increases reflectance of photoreceptor outer segments.	LLRL was safe over 12 months.
Yang et al. 2025 [37]	Cycloplegic SER: −1.00 to −1.00 D	The LLRL device emitted at 650 nm with a power of 0.39 mW.	1.00	AL was shorter in the LLRL group.	No adverse event	LLRL for 12 months showed reduced myopia progression.
Xu et al. 2024 [38]	Cycloplegic SER: −4.00 D or more	650 nm that delivers 1600 lx of LLRL through the pupil with a power of 0.29 mW.	1.00	SER change after 12 months was 0.11 D and −0.75 D in the intervention and control groups, respectively.	Conjunctivitis	LLRL therapy is effective in high myopia, with 53.3% showing notable axial shortening.
Zhang et al. 2025 [39]	Cycloplegic SER: −0.25 to −6.00 D	LLRL therapy used a desktop device emitting 650 ± 10 nm red light at 0.12 mW through a 4 mm pupil. Sessions lasted 3 min, twice daily, a 4 h, for 5 days per week.	2.00	SER progression and AL elongation were significantly lower in the LLRL group than in the LDA group at 6 months (SER: *p* = 0.002; AL: *p* = 0.004), and at both 12 and 24 months (*p* < 0.001).	Eight adverse events occurred only in the LLRL group: one allergic reaction, four cases of altered color perception, one conjunctivitis, and two cases of dry eye.	LLRL is more effective than 0.01% atropine in slowing myopia progression and shows a higher proportion of children with SER regression and AL shortening over two years.
Xiang et al. 2025 [40]	Cycloplegic SER: −0.5 D or more	LLRL group received 3 min sessions twice daily, five days a week, 4 h between sessions.	1.00	CT increased significantly in LLRL group.	No adverse event	LLRL treatment increased CT in pre-myopic children, especially in the subfoveal region.
Zhang et al. 2025 [41]	Cycloplegic SER: −1.00 and +1.00 D	Participants received twice-daily LLRL therapy using 650 nm.	1.00	AL elongation increased by 0.05 mm in the LLRL group and 0.25 mm in the control group (*p* < 0.001). SER progression was −0.20 D in the LLRL group and −0.61 D in the control group (*p* < 0.001).	Three eyes in the LLRL group showed transient dome-shaped hyperreflectivity between the RPE and the foveal ellipsoid zone.	The importance of routine OCT scans during LLRL therapy for detecting retinal changes.
Chen et al. 2026 [42]	Not reported	LLRL therapy using a 650 nm (3 min/session, ≥4 h interval), 5–7 days/week.	3.00	(Annual AL elongation ≤0.10 mm) was 72.53% over more than 3 years with an average AL growth of 0.06 mm per year.	A minimal and reversible retinal change was observed in four eyes	Long-term efficacy and safety in myopia control over 3 years.
Lili et al. 2025 [43]	Cycloplegic SER	LLRL using a 650 nm desktop device, twice daily for 3 min, 5 days per week.	2.00	Over 2 years, 65.5% achieved complete myopia control based on AL elongation (<0.10 mm/year), and 68.2% based on SER progression (<0.25 D/year).	No adverse event	About 70% of children achieved complete myopia control over 2 years.
Liao et al. 2025 [44]	Cycloplegic SER: Less than−6.00 D	LLRL devices operating at 0.2 to 2 mW laser power.	1.00	Reduced cone density within 0.5 mm of the foveal center, especially in the temporal area.	Developed small cystoid changes in the ganglion cell layer	Reduced paracentral foveal cone density and subtle retinal changes.

D = Diopters, AL = Axial length, SER = Spherical equivalent refraction, SVS = Single-vision spectacle, SFCT = Subfoveal choroidal thickness, LDA = low-dose atropine, RPE = Retinal pigment epithelia, CT = Choroidal thickness.

**Table 5:** Studies used short-wavelength light therapy for myopia control and prevention.

Author and Year	Myopia Severity	Details of Intervention	Follow Up (Year)	Outcome Measure	Safety	Effectiveness
Mori et al. 2021 [45]	Cycloplegic SER:−1.50 to −4.5 D	VL-transmitting eyeglasses allow 360–400 nm VL to pass through.	2.00	AL in the VL glasses group was significantly smaller than in placebo glasses group.	No adverse effects	VL glasses group showed 21.4% decrease in AL elongation over 2 years.
Torii et al. 2022 [46]	Cycloplegic SER: −1.50 to −4.5 D	Ultraviolet transmitting eyeglasses allow 310 nm VL for 3 h.	0.5	Significant changes were seen in AL elongation, choroidal thickness, and SER.	No adverse effects	Ultraviolet glasses showed short-term safety and effectiveness in slowing myopia progression.
Mehler et al. 2025 [47]	Cycloplegic SER: −0.64 ±1.23 D	A one-minute 450 nm blue light stimulus was applied to the optic disk or central retina.	0.5	No significant changes in choroidal thickness were found between myopic and non-myopic participants.	No adverse effects	Short-duration blue light stimulation of the optic disk is ineffective for activating retinal dopamine pathways to prevent myopia.
Thakur et al. 2021 [48]	−1.75 to −7.00 D	Blue (460 nm), green (521 nm), red (623 nm), and white light for one hour each across four separate sessions on different days.	-	Blue light exposure reduced AL in both eyes (−8.0 ± 3 µm, *p* < 0.001 in the defocused eye; −6.0 ± 3 µm, *p* = 0.11 in the non-defocused eye), with no significant change in choroidal thickness.	No adverse effects	Red and green light caused AL elongation, while blue light inhibited AL elongation in human eyes.
Chakraborty et al. 2024 [49]	Cycloplegic SER −3.12 ± 2.97 D	Participants completed 2 h sessions of cyan, red, white, and blue light on separate days using light-emitting glasses.	2 h	The effects of cyan light on AL and CT were found to be stronger in the myopic eye.	No adverse effects reported	Exposure to cyan light reduced AL and increased CT in both young adults and children.
Wu et al. 2023 [50]	Not reported −0.5 to −6.0 D	Participants completed all four interventions: 0 D, 1 D, and 2 D defocus, and 1 D defocus with 30% blue light filtering.		Both 1 D and 2 D defocus are associated with increased CT.	No adverse effects reported	Blue light defocus technology may reduce hyperopic defocus, improve retinal perfusion, and ease visual fatigue, helping myopia prevention.

D = Diopters, AL = Axial length, SER = Spherical equivalent refraction, LDA = low-dose atropine, RPE = Retinal pigment epithelial, UV = ultraviolet, VL = violet light.

**Table 6:** Comparative summary of LLRL and short-wavelength light therapies for myopia control and prevention.

Feature	LLRL Therapy	Short-Wavelength Light Therapy
**Typical follow-up duration**	Two months to 3 years (most studies 6 to12 months; long-term up to 3 years)	One hour to 2 years (most studies 6 months to 2 years)
**Outcome measures**	AL, SER, choroidal thickness, retinal thickness and myopia incidence	AL, CT, SER, retinal response measures, light exposure metrics
**Mean AL reduction (approximate)**	Reduction of approximately 0.10–0.30 mm per year compared with controls	Reduction is smaller (approximately 0.02–0.10 mm per year), and some studies report no significant change
**Mean SER (approximate)**	+0.05 to +0.25 D/year (vs. −0.25 to −0.79 D in controls)	Minimal improvement; small shifts or stabilization, often not statistically significant
**Mean choroidal thickness change**	Increase approximately 5–45 µm (notably subfoveal CT across studies)	Increase approximately 5–10 µm transiently after blue/violet light exposure
**Effectiveness**	Strong and consistent reduction in myopia progression; effective for prevention and control	Moderate and variable; wavelength- and exposure-dependent effects
**Safety**	Potential safety concerns underscore the need for further long-term safety evaluation of LLRL therapy	No adverse effects were reported in most studies; further long-term studies are required to establish the safety of the therapy
**Strength of evidence**	High (multiple clinical trials including long-term studies)	Moderate to low (mixed experimental and clinical data)
**Clinical applicability**	Promising therapeutic option, although questions regarding long-term safety and generalizability remain concerns.	Emerging adjunct/preventive strategy

## Data Availability

All data supporting the findings of this systematic review are available within the paper in Table 1, Table 2, Table 3 and Table 4 and in the original references used in this study.

## Supplementary Materials

The following supporting information can be downloaded at https://www.mdpi.com/article/10.3390/jcm15218493/s1: Table S1: The PRISMA 2020 checklist.
